# Effect of Irrigation with Zirconia Microparticles Combined with Different Activation Techniques on Smear Layer Removal: An Ex Vivo Study

**DOI:** 10.3390/dj14080497

**Published:** 2026-08-07

**Authors:** Hussein Imad Al-Ghurairi, Hussain Al-Huwaizi

**Affiliations:** Department of Esthetic and Restorative Dentistry, College of Dentistry, University of Baghdad, Bab Al-Muadham, Baghdad P.O. Box 1417, Iraq; hussein.imad@ibnsina.edu.iq

**Keywords:** Er,Cr:YSGG laser, passive ultrasonic irrigation, smear layer removal, Zirconia microparticles

## Abstract

**Background/Objective:** Root canal debridement is limited by complex anatomy and the apical vapor lock phenomenon, reducing smear layer removal. This study evaluated the effect of a 5 µm zirconia microparticle suspension used with sonic, passive ultrasonic irrigation (PUI), or erbium, chromium-doped yttrium scandium gallium garnet (Er,Cr:YSGG) laser activation on smear layer removal across the root canal thirds. **Methods:** Sixty single-rooted human teeth were prepared to size F4 (40/0.06) using 5.25% NaOCl and randomly assigned to six groups (*n* = 10) combining activation modality (sonic, PUI, or Er,Cr:YSGG laser) and irrigant type (distilled water or 5 µm zirconia suspension) over a 5 min interrupted cycle. Smear layer removal was scored using scanning electron microscopy (SEM) and the Hülsmann grading system. Data were analyzed via Kruskal–Wallis and Friedman tests with post hoc pairwise comparisons (α = 0.05). **Results:** Inter-rater reliability was strong (Cohen’s κ = 0.736–0.822; *p* < 0.001). Smear layer removal varied significantly across root thirds (*p* < 0.001). Ultrasonic + Zirconia demonstrated superior removal in the middle third compared with Sonic + Distilled Water (*p* = 0.005), and in the coronal third compared with Laser + Distilled Water (*p* = 0.003) and Sonic + Distilled Water (*p* = 0.026). Coronally, Ultrasonic + Distilled Water outperformed Laser + Distilled Water (*p* = 0.041). No significant differences occurred apically (*p* > 0.05). **Conclusions:** Incorporating 5 µm zirconia microparticles with passive ultrasonic activation improved smear layer removal, particularly in the coronal and middle thirds of the root canal under the conditions of this ex vivo study.

## 1. Introduction

Complete eradication of intracanal microorganisms is critical for the long-term success of root canal therapy, as persistent microbial infection is the primary etiological factor associated with apical periodontitis and post-treatment endodontic disease [1]. Although mechanical instrumentation effectively shapes the main root canal space, it cannot completely instrument the complex root canal anatomy, leaving substantial areas of the canal walls uninstrumented [2]. Consequently, anatomical features such as fins, isthmuses, recesses, and lateral canals can continue to harbor pathogenic microorganisms. Effective irrigation protocols are therefore necessary to access these complex areas, optimize debridement and disinfection, and improve treatment outcomes [3]. Modern endodontics utilizes advanced thermomechanically treated nickel–titanium (NiTi) rotary systems, including Blue and Gold wire technologies. These metallurgical advancements improve file flexibility, cyclic fatigue resistance, and shape memory by altering crystalline phase transition pathways. The resulting martensitic behavior facilitates more centered canal preparation and enables instruments to negotiate severe curvatures while reducing the risk of intracanal instrument separation [4,5]. However, friction generated between these flexible instruments and dentinal walls inevitably results in smear layer formation. This amorphous layer comprises bacteria and their byproducts, organic tissue remnants, and inorganic dentin particles. By coating canal walls and occluding dentinal tubule orifices, the smear layer can impair sealer adaptation, reduce irrigant and intracanal medication penetration, and compromise root canal disinfection [6,7]. While manual syringe irrigation remains the conventional clinical standard, quantitative computational fluid dynamics (CFD) models demonstrate inherent physical limitations. Conventional irrigation exhibits deficient hydrodynamic exchange, restricting irrigant replacement to approximately 1–2 mm beyond the needle tip and leaving the apical third stagnant. Furthermore, the closed-end root canal anatomy frequently traps ambient gases at the apex. This phenomenon, termed the apical vapor lock effect, acts as a physical gas barrier that prevents liquid irrigants from reaching, debriding, or chemically interacting with the apical third [8,9]. Consequently, conventional liquid-only irrigation protocols often fail to completely debride the intricate anatomy of the apical region because of vapor lock formation and limited fluid exchange. These limitations necessitate improving irrigation efficacy while minimizing the chemical erosion and structural alterations that may result from traditional demineralizing agents. Accordingly, contemporary endodontics has shifted toward advanced irrigation activation technologies that enhance fluid dynamics, improve irrigant distribution, and promote mechanical debridement of the root canal system [10,11]. Incorporating abrasive microparticles into irrigant solutions introduces a mechanical scouring mechanism that removes the smear layer through physical abrasion rather than chemical demineralization [12]. Effective dispersion of these particles requires activation technologies. Sonic systems agitate irrigants using flexible polymer tips operating at frequencies of approximately 1–10 kHz, whereas passive ultrasonic irrigation (PUI) operates at 25–30 kHz, generating acoustic streaming and cavitation that enhance irrigant exchange and promote particle transport into anatomically inaccessible regions of the root canal system. Additionally, Er,Cr:YSGG (2780 nm) laser-activated irrigation provides a photomechanical approach in which strong water absorption induces rapid vapor bubble expansion and collapse, generating shockwaves that enhance particle–wall interactions and mechanical debridement of canal surfaces [13,14]. Mechanical particle-assisted debridement operates through the transfer of fluid kinetic energy from the irrigant to suspended microparticles, enabling particle transport and interaction with dentinal surfaces during activation [8,15]. From a tribological perspective, abrasive particles function as third bodies interposed between the irrigant and canal walls, with their effectiveness influenced by particle size, morphology, and hardness [16]. Experimental studies demonstrate that ultrasonic activation generates distinct kinetic energy distributions around the file tip, with turbulent energy increasing above a critical power threshold, potentially facilitating effective particle–dentin interactions [17]. Furthermore, abrasive particle supplementation has been shown to improve ultrasonic irrigation efficacy for smear layer removal [12,18]. Collectively, these findings provide the scientific rationale for investigating zirconia microparticles as a mechanical adjunct to irrigation activation. However, the smear layer removal efficacy of 5 µm zirconia microparticles activated by sonic, passive ultrasonic, or Er,Cr:YSGG laser methods has not been evaluated across the coronal, middle, and apical thirds of the root canal. To isolate the mechanical contribution of zirconia microparticles, a baseline comparison using an inert vehicle was methodologically essential. Distilled water was selected as the control irrigant rather than a chelating agent (e.g., 17% EDTA) to isolate the mechanical effects of zirconia microparticles and irrigation activation while avoiding the confounding influence of chemical demineralization. Therefore, this ex vivo study aimed to evaluate the smear layer removal efficacy of a 5 µm zirconia microparticle solution compared with a baseline distilled water control under these activation modalities using scanning electron microscopy (SEM). The null hypothesis (H_0_) stated that no statistically significant differences in smear layer removal would exist among the experimental groups at the apical, middle, and coronal thirds.

## 2. Materials and Methods

### 2.1. Study Design and Sample Selection

This ex vivo study evaluated the effectiveness of a 5 μm zirconia microparticle suspension activated by different irrigation techniques for smear layer removal under controlled laboratory conditions. Research ethics approval (Approval no: 1072; Date: 13 November 2025) was obtained from the Research Ethics Committee of the College of Dentistry, University of Baghdad, where the study was conducted. Informed consent was unnecessary for this research since it was an ex vivo analysis using extracted human teeth obtained in compliance with conventional clinical practices. Sixty freshly extracted human single-rooted teeth from individuals aged 18–50 years were included. Inclusion criteria comprised straight roots with circular cross-sections, mature apices, and no evidence of caries, resorption, or previous endodontic treatment. Following calculus removal and pumice polishing, teeth were stored in 0.1% thymol solution [19]. To ensure statistical adequacy, an a priori sample size calculation was performed using G*Power (version 3.1.9.7). An effect size (f = 0.50) was assumed based on a previous study evaluating active irrigation protocols [20]. With α = 0.05 and 80% power across six groups, the minimum required sample size was 54 specimens (9 per group). In order to improve statistical reliability and account for possible specimen loss during testing, 60 specimens (10 per group) were included.

### 2.2. Sample Preparation and Instrumentation

A digital electronic caliper was used to standardize the root length of each specimen to 14 mm. A diamond disc was used to segment the specimens perpendicular to the long axis under continuous water cooling. A size 10 K-file was used to navigate the canal until the tip was visible at the apical foramen, at which point 0.5 mm was removed to achieve the working length (WL). Specimens were vertically aligned using a dental surveyor and placed in silicone putty within plastic tubes. Gates-Glidden drills, sizes one and two, were used for coronal flaring. A nickel–titanium rotary instrument system (Lusterdent Pliancy Blue/Gold; Lusterdent Medical Instrument Co., Zhengzhou, China) was used to finish the biomechanical preparation from SX to F4 (40/0.06) at 300 rpm and 2.0 N·cm torque. Each rotary file set was utilized for a maximum of five canals. A 30-gauge side-vented needle positioned 2 mm short of the WL was used during instrumentation to irrigate the canals with 1 mL of 5.25% NaOCl in between file changes.

### 2.3. Experimental Grouping and Activation Protocols

Following crown sectioning, the 60 specimens were placed in an opaque container, thoroughly mixed, and randomly assigned by sequential blind drawing into three main experimental groups according to activation modality (n = 20 each): Group A (sonic activation), Group B (passive ultrasonic activation), and Group C (Er,Cr:YSGG laser activation). Each main group was subsequently subdivided into two subgroups (n = 10 each) using the same sequential blind-drawing procedure according to irrigant type: distilled water (control; A1, B1, C1) or distilled water supplemented with 5 µm zirconia microparticles (experimental; A2, B2, C2). During both randomization stages, specimens were drawn individually by a research assistant who was not involved in specimen preparation or SEM evaluation, without visual selection, to minimize allocation bias. The randomization sequence remained concealed from the operators performing the activation protocols and the SEM examiners until completion of data collection. For each root canal assigned to the experimental subgroups, 50 mg of zirconium oxide microparticles (nominal diameter: 5 µm; monoclinic crystalline structure; irregular morphology; Sigma-Aldrich, St. Louis, MO, USA) were suspended in 10 mL of distilled water to prepare the working suspension. The reported physicochemical characteristics were based on the manufacturer’s specifications, as independent characterization was not performed. Particle sedimentation was observed before use; therefore, the suspension was manually agitated immediately prior to administration to redisperse the particles. To standardize irrigant activation and minimize heat generation, an interrupted cycle method was used across all groups. Each root canal received a total of 10 mL of irrigant, administered in 2 mL increments: distilled water in the control subgroups (A1, B1, and C1) and the zirconia-containing suspension in the experimental subgroups (A2, B2, and C2). The total activation period for each canal was 5 min, with each 2 mL increment being activated for 1 min and then resting for 10 s. Ten-milliliter disposable syringes were used to administer each irrigant at a controlled flow rate of 0.3 mL/s. To ensure the removal of residual zirconia particles, the canals in the experimental subgroups (A2, B2, and C2) received an additional cleaning stage after the activation treatment. This involved a final flush of 5 mL of distilled water administered in 1 mL increments, each of which was activated for 1 min using the same interrupted cycle technique.

The device-specific parameters are as follows.

• Sonic activation (A1, A2): A sonic activation device (Eighteeth, Changzhou, China) equipped with a medium polymer tip (25/0.04) was operated in high mode according to the manufacturer’s instructions.
• Ultrasonic Activation (Groups B1, B2): The ultrasonic activation device (Ultra X; Eighteeth, China) operated in High Output Power Mode (45 kHz) utilizing a stainless-steel tip (B18, 20/0.02).
• Laser Activation (Groups C1, C2): An erbium, chromium-doped yttrium scandium gallium garnet (Er,Cr:YSGG) laser system (Waterlase iPlus; Biolase, Foothill Ranch, CA, USA) was used with a radial firing tip (RFT3; Biolase) at 1.25 W and 50 Hz.

Cementoenamel temperature was monitored using an infrared thermal camera (UTi716S; UNI-T, Dongguan, China) and showed an increase of approximately 2 °C, indicating thermal safety.

### 2.4. Root Sectioning and Sample Preparation

The root canals were dried using sterile paper points in accordance with the experimental procedures. The roots were removed from their silicone mounting blocks to enable longitudinal splitting for internal surface examination. A diamond disc (22 × 0.4 mm; Komet Dental, Lemgo, Germany) set on a low-speed handpiece under constant water cooling was used to create two parallel longitudinal orientation grooves on the buccal and lingual surfaces of each root. The grooves were carefully formed without penetration into the root canal space to ensure that the internal canal shape remained intact and to avoid the introduction of exogenous debris. To protect the dentinal walls throughout the splitting process, a gutta-percha point of size 40/0.06 was placed inside the canal as an internal physical barrier. The root was then divided into two symmetrical sections by placing a No. 11 surgical blade into the created grooves and tapping it lightly with a hammer. One half was randomly assigned for scanning electron microscopy (SEM) evaluation.

### 2.5. Scanning Electron Microscopy Evaluation

To assess the residual smear layer, one half of each split root was prepared for scanning electron microscopy (SEM). The samples were fixed for 12 h at 4 °C in a 0.1 M sodium cacodylate buffer (BDH Chemicals Ltd., Poole, England; pH 7.4) with 2.5% buffered glutaraldehyde (EOBA Chemie Pvt., Mumbai, India). The samples were rinsed with distilled water for an hour after fixation, with the water being changed every 20 min. Dehydration was accomplished using a graded ethanol series (25%, 50%, and 75% for 20 min each; 95% for 30 min; and 100% for 60 min). The samples were vacuum-sputter-coated with gold after being air-dried for a whole day on aluminum stubs.

An SEM system (TESCAN VEGA III; TESCAN, Brno, Czech Republic) was used to inspect the interior surfaces. The magnification of the micrographs was 1000×. Measurements were performed at standardized distances from the root apex. Three levels were assessed: apical (1–3 mm), middle (6–8 mm), and coronal (10–12 mm). Imaging was standardized at three locations within each level: one in the center of the corresponding third, and two additional points located 1 mm above and 1 mm below the center. Two blinded, calibrated observers assessed smear layer presence using the 4-point Hülsmann grading system, with scores defined as follows: Score 1, no smear layer with completely open dentinal tubules; Score 2, a thin smear layer with most tubules open; Score 3, a homogeneous smear layer covering the canal wall with few open tubules; and Score 4, a complete smear layer covering the entire wall with no open tubules. When observers disagreed, the specimens were reassessed until a consensus was reached.

### 2.6. Statistical Analysis

IBM SPSS Statistics version 23.0 (IBM Corp., Armonk, NY, USA) was used to analyze the data. Statistical significance was set at *p* < 0.05. Cohen’s kappa (κ) was used to evaluate inter-rater reliability for smear layer scoring on 60 samples. The Kruskal–Wallis test was used to compare groups within each anatomical third for the non-parametric smear layer scores, and Friedman’s two-way ANOVA by ranks was used to evaluate regional differences within the same tooth. For post hoc analysis, pairwise comparisons with Bonferroni correction were employed.The minimal dataset and accompanying README describing the variables used for the statistical analyses are provided in the Supplementary Materials (Dataset S1 and README S1).

## 3. Results

### 3.1. Inter-Rater Reliability

Cohen’s kappa (κ) demonstrated strong inter-examiner agreement across all root regions. The apical third showed the highest agreement (κ = 0.822), whereas the middle (κ = 0.751) and coronal thirds (κ = 0.736) demonstrated substantial agreement (*p* < 0.001), confirming the reproducibility of the scoring method. (Table 1).

### 3.2. Smear Layer Removal

Smear layer removal scores for the six irrigation protocols are presented as median values and interquartile ranges (IQR). An anatomical gradient was observed across all groups, with the apical third showing the lowest cleaning efficacy and the coronal third the greatest smear layer removal (lowest scores). The Ultrasonic + Zirconia group achieved the lowest median smear layer score in the coronal third (median = 2.00), whereas the Sonic + Distilled Water group consistently exhibited a median score of 4.00. Significant differences among the six groups were observed in the apical (*p* = 0.007), middle (*p* = 0.002), and coronal thirds (*p* = 0.001) (Kruskal–Wallis test). Post hoc pairwise comparisons with Bonferroni correction were performed to identify differences between the irrigation protocols. The descriptive statistics are presented in Table 2.

**Figure 1:**
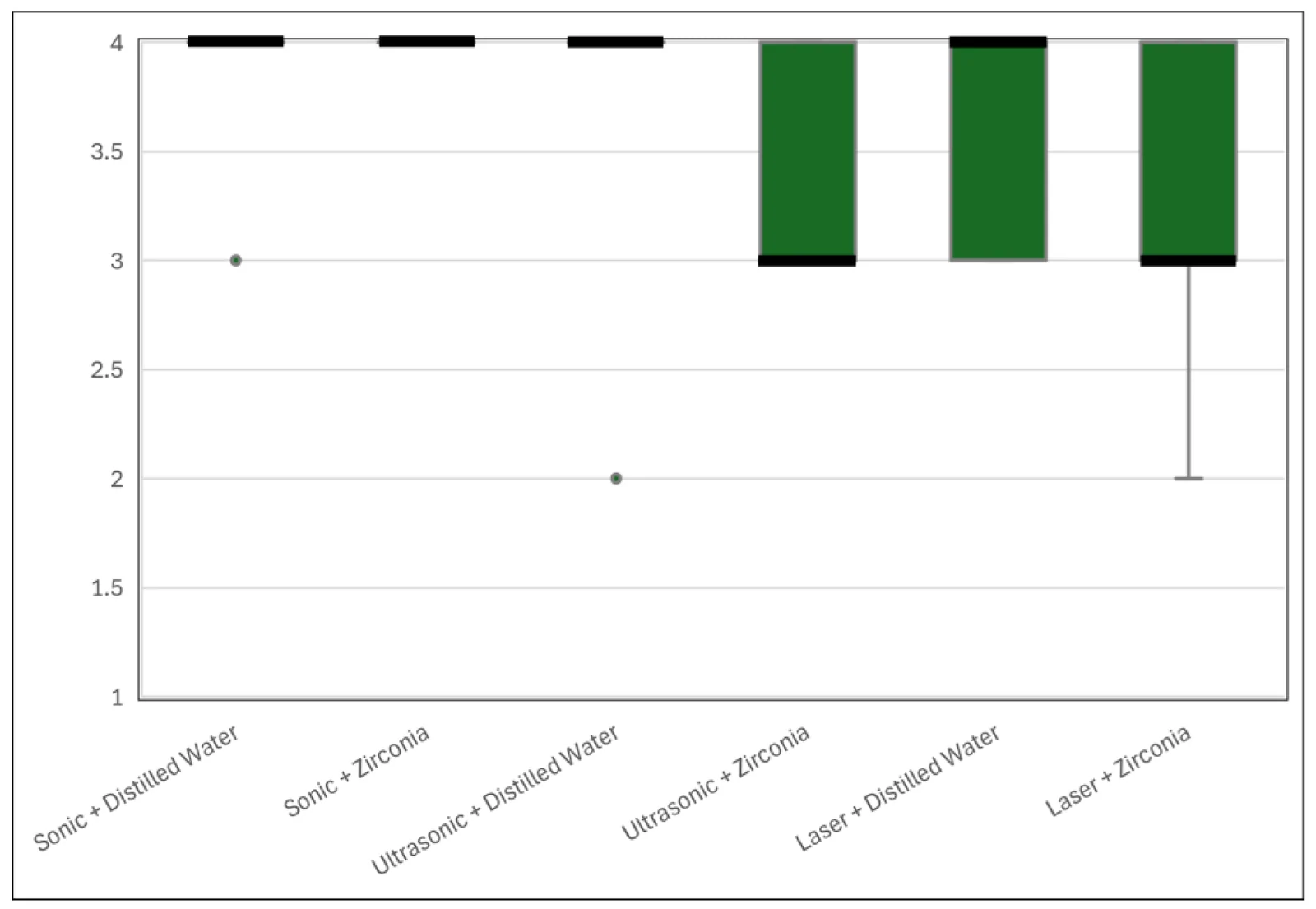
Box-and-whisker plot showing smear layer removal scores in the apical third (n = 10). The black horizontal line within each box represents the median score, while the box height indicates the interquartile range (IQR). Whiskers represent the data range, and points denote outliers. A single black horizontal line indicates complete score uniformity (IQR = 0).

Although the overall Kruskal–Wallis test was significant, adjusted pairwise comparisons for the apical third (Figure 1) revealed no statistically significant differences between individual groups (*p* > 0.05).

**Figure 2:**
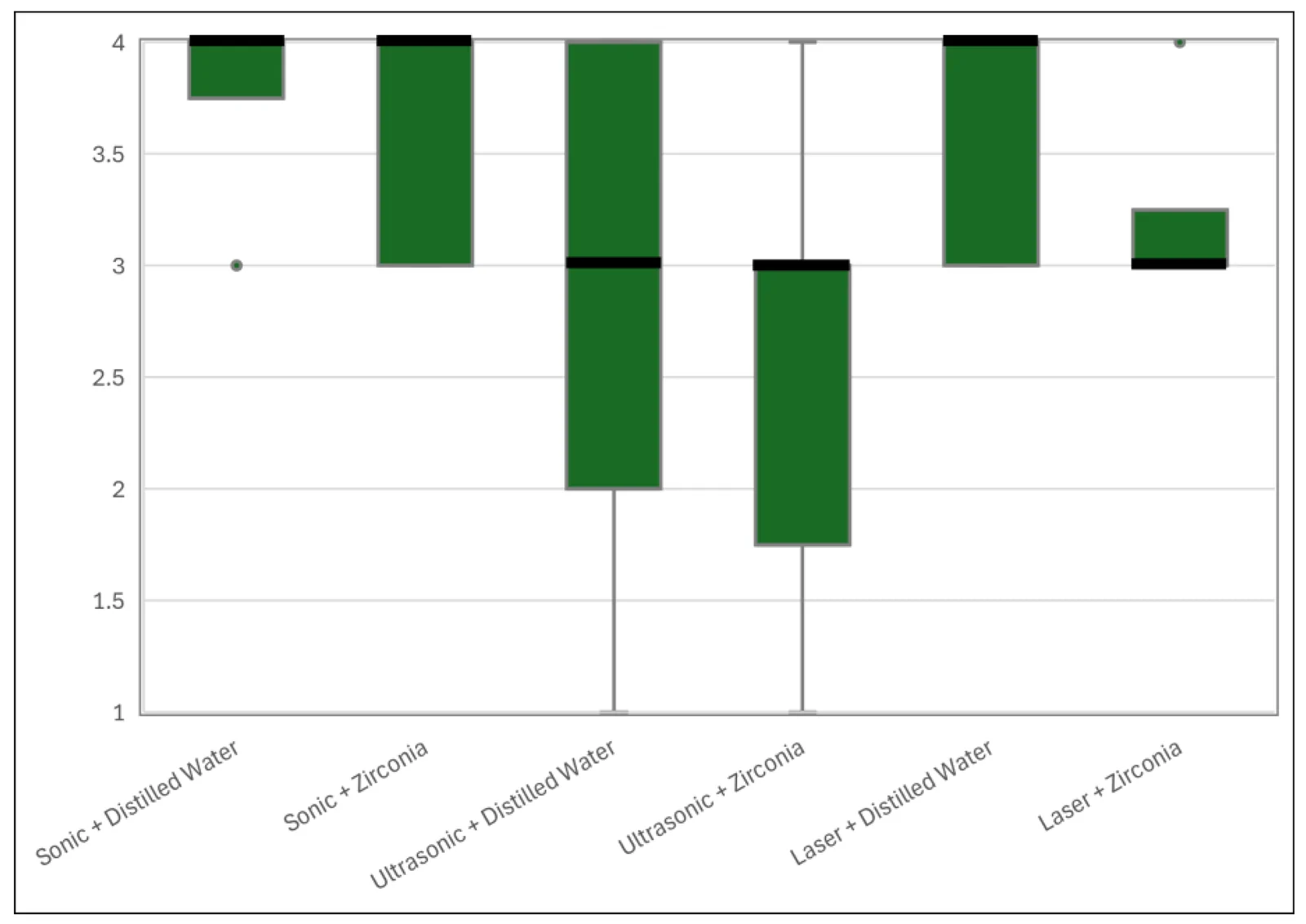
Box-and-whisker plot showing smear layer removal scores in the middle third (n = 10). The dots represent outliers in the box-and-whisker plots.

**Figure 3:**
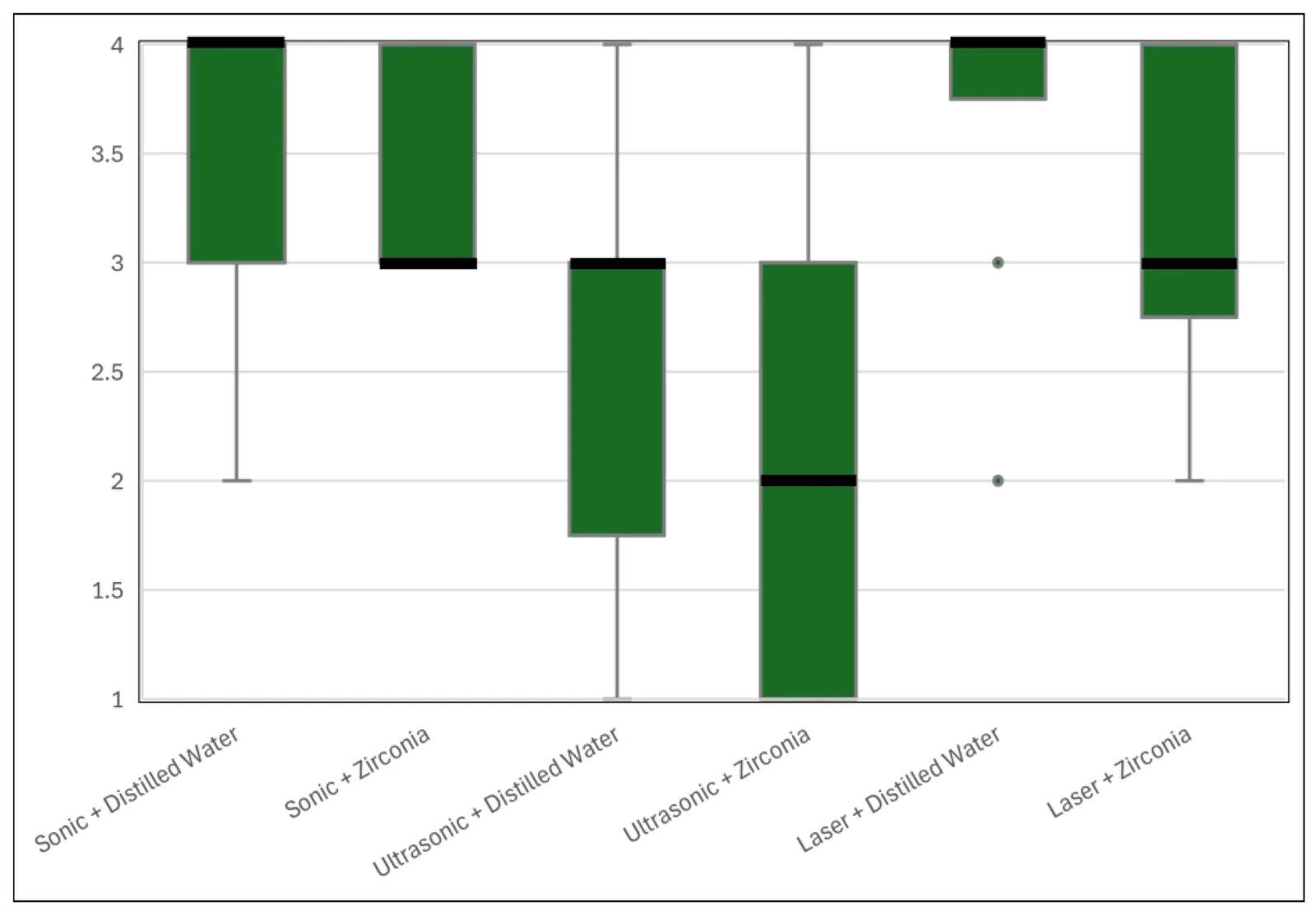
Box-and-whisker plot showing smear layer removal scores in the coronal third (n = 10). The dots represent outliers in the box-and-whisker plots.

• In the middle third (Figure 2), Ultrasonic + Zirconia significantly exceeded the Sonic + Distilled Water control in the field of smear layer reduction (*p* = 0.005).

• In the coronal third (Figure 3), Ultrasonic + Zirconia performed better than both Laser + Distilled Water (*p* = 0.003) and Sonic + Distilled Water (*p* = 0.026). Additionally, Ultrasonic + Distilled Water showed superior performance compared to Laser + Distilled Water (*p* = 0.041).

**Figure 4:**
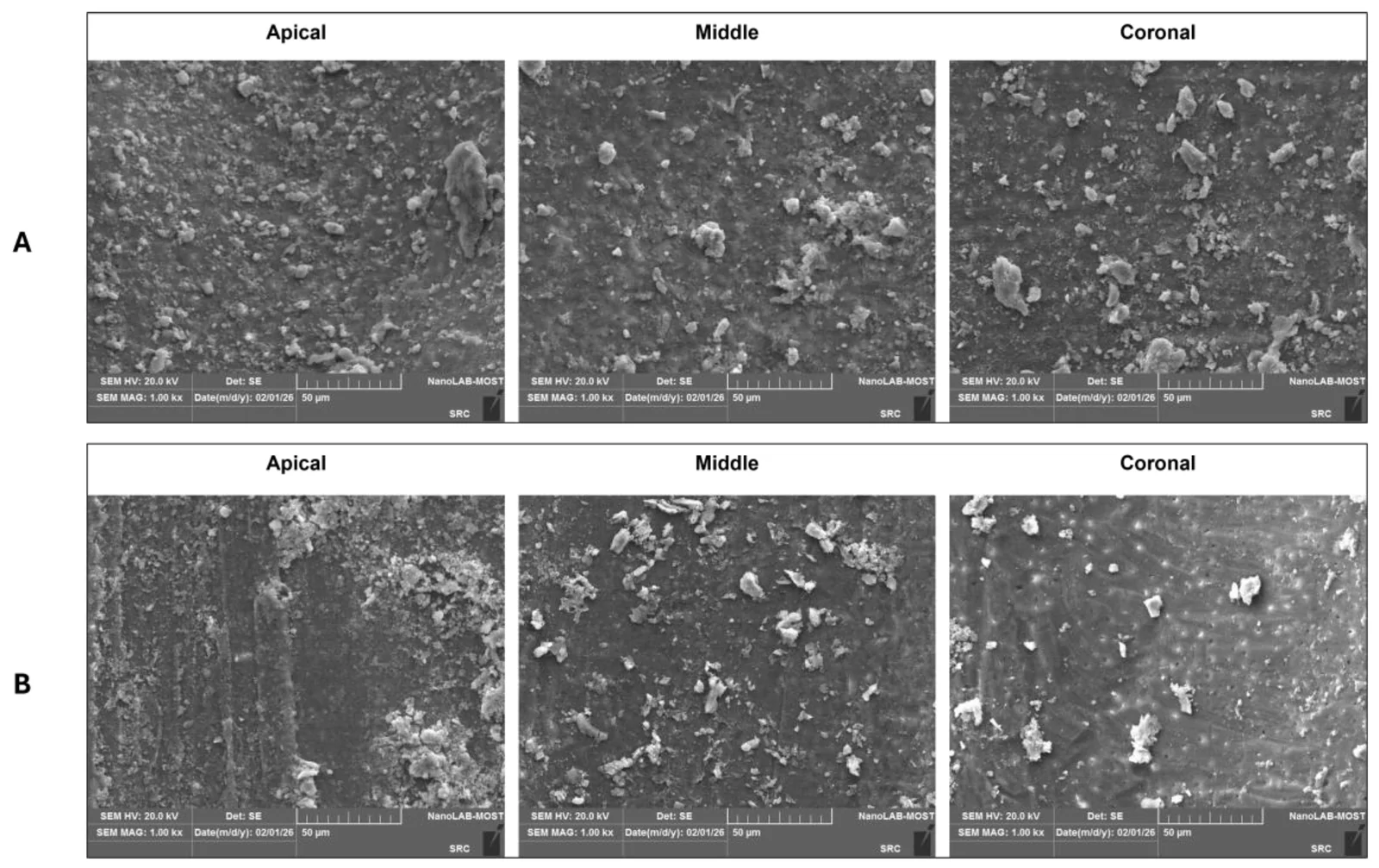
Representative (SEM) micrographs of root canal dentin following sonic activation (1000× magnification). (**A**) Group A1 (Sonic + distilled water): dense, homogeneous smear layer completely covering the canal walls with no visible dentinal tubule openings (Hülsmann score 4) in the apical, middle, and coronal thirds. (**B**) Group A2 (Sonic + zirconia): dense, homogeneous smear layer with no visible dentinal tubule openings in the apical and middle thirds (Hülsmann score 4), and a thinner smear layer with limited visible dentinal tubule openings in the coronal third (Hülsmann score 3).

**Figure 5:**
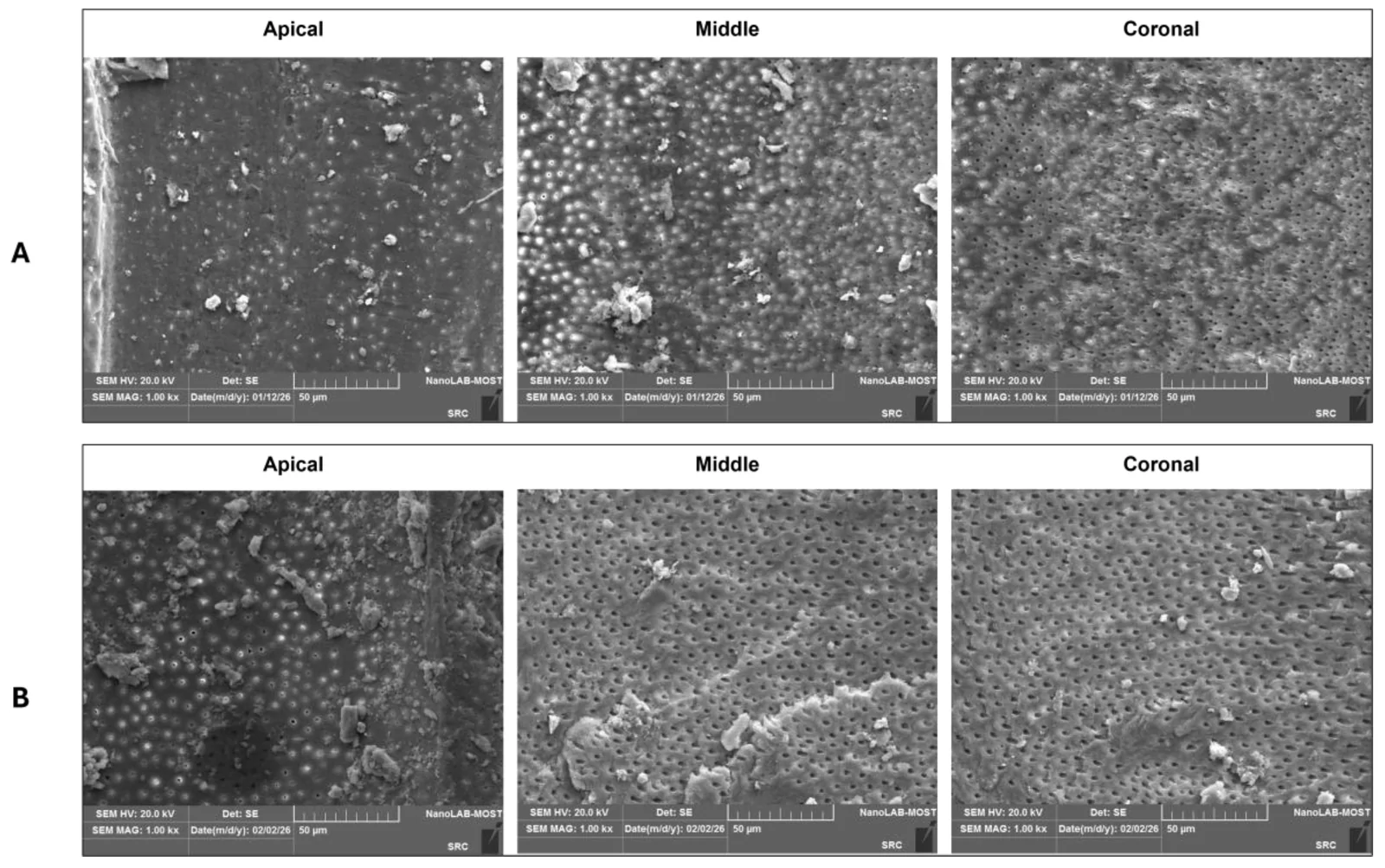
Representative SEM micrographs of root canal dentin following passive ultrasonic activation (1000× magnification). (**A**) Group B1 (Ultrasonic + distilled water): dense, homogeneous smear layer completely obscuring dentinal tubule openings in the apical third (Hülsmann score 4) and a homogeneous smear layer with limited visible tubule openings in the middle and coronal thirds (Hülsmann score 3). (**B**) Group B2 (Ultrasonic + zirconia): homogeneous smear layer with limited visible dentinal tubule openings in the apical and middle thirds (Hülsmann score 3), and minimal smear layer with many visible dentinal tubule openings in the coronal third (Hülsmann score 2).

**Figure 6:**
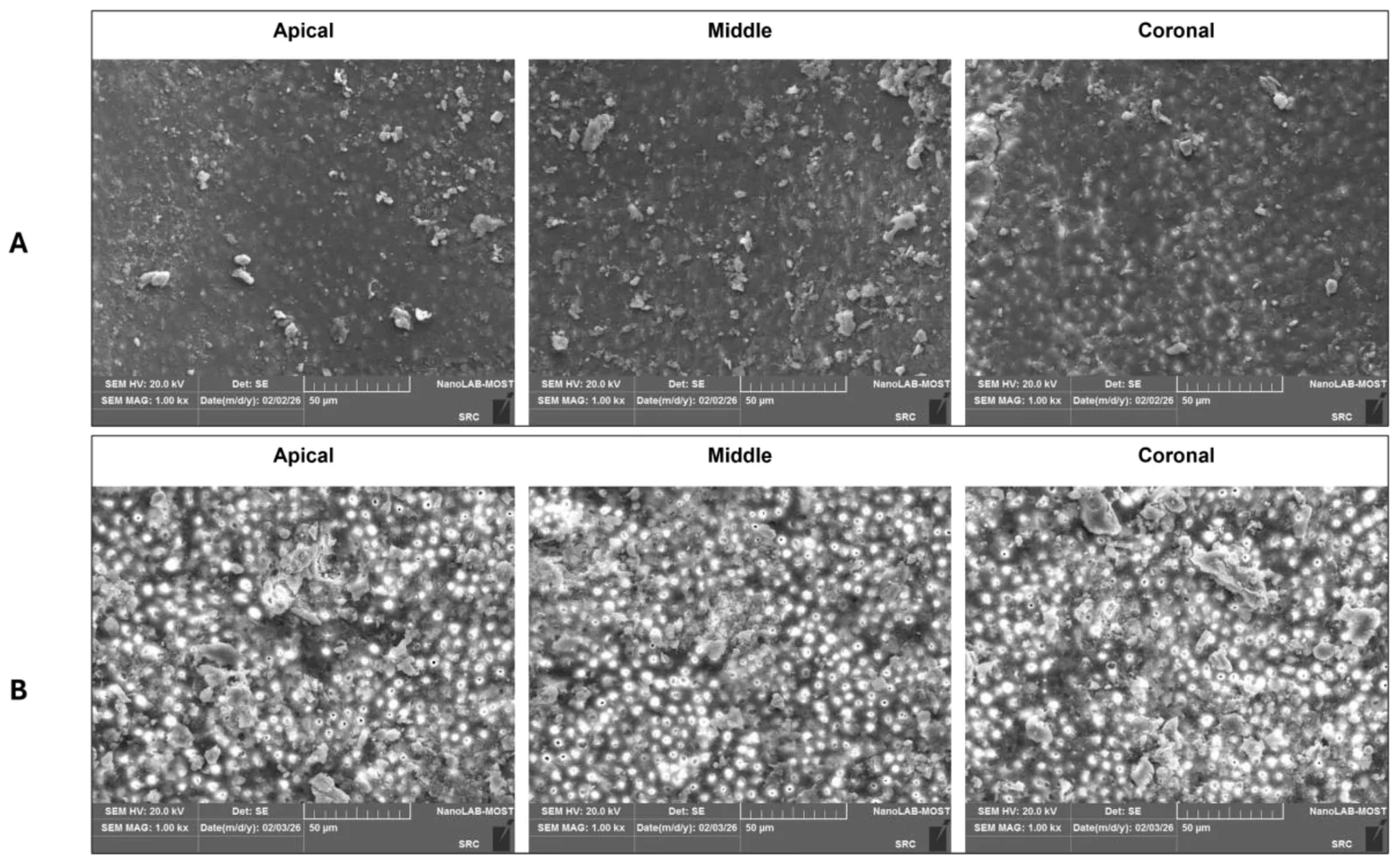
Representative SEM micrographs of root canal dentin following Er,Cr:YSGG laser activation (1000× magnification). (**A**) Group C1 (Er,Cr:YSGG laser + distilled water): dense, homogeneous smear layer covering the canal walls with no visible dentinal tubule openings in the apical, middle, and coronal thirds (Hülsmann score 4). (**B**) Group C2 (Er,Cr:YSGG laser + zirconia): homogeneous smear layer with limited visible dentinal tubule openings across the apical, middle, and coronal thirds (Hülsmann score 3).

Furthermore, Friedman’s ANOVA confirmed that smear layer removal scores differed significantly across the three anatomical locations (*p* < 0.001). Subsequent post hoc analysis demonstrated that smear layer removal was significantly greater in the coronal third than in the apical third (*p* = 0.001). These regional cleaning patterns are qualitatively demonstrated in the representative SEM micrographs (Figure 4, Figure 5 and Figure 6).

## 4. Discussion

The present study demonstrated that both the activation technique and the incorporation of 5 µm zirconia microparticles influenced smear layer removal and radicular surface cleanliness. Accordingly, the null hypothesis was rejected. Passive ultrasonic activation combined with zirconia microparticles produced the greatest smear layer removal, particularly in the coronal and middle thirds, as assessed by SEM. Although these findings are supported by the experimental data, the proposed mechanisms involving tribology, fluid dynamics, particle transport, and hydrokinetic interactions remain theoretical because they were not directly investigated.

### 4.1. Mechanistic Basis for Smear Layer Removal

The observed cleaning efficiency hierarchy (Ultrasonic > Laser > Sonic) stems from distinct fluid dynamics and energy transmission mechanisms. Passive ultrasonic irrigation (PUI) achieves superior smear layer removal through the generation of intense acoustic streaming and secondary microcurrents induced by high-frequency oscillation of the irrigant. These hydrodynamic phenomena significantly increase shear stress along dentinal walls and enhance irrigant penetration into canal irregularities, thereby promoting effective disruption and removal of the smear layer. Recent in vitro evidence further confirms that ultrasonic activation enhances dentinal surface debridement through improved fluid dynamics and continuous replenishment of the irrigant within confined canal spaces [15,22]. This effect may have been augmented by the incorporation of 5 µm zirconia microparticles. Although the underlying mechanism was not directly investigated, suspended zirconia particles may increase mechanical interaction between the activated irrigant and dentinal surfaces, thereby contributing to the observed improvement in smear layer removal [12].

In contrast, Er,Cr:YSGG laser activation removes debris through photomechanical effects, including rapid vapor bubble formation, expansion, and collapse, which generate localized cavitation and acoustic streaming within the irrigant. However, this mechanism occurs in a pulsed and intermittent manner, which may limit the continuity of fluid agitation and irrigant exchange when compared with the sustained acoustic streaming produced by passive ultrasonic irrigation [23,24]. Lower-frequency sonic activation demonstrated reduced smear layer removal compared with ultrasonic and laser activation, particularly in the apical third, where tip damping, energy dissipation, and restricted irrigant penetration limit hydrodynamic efficiency [3,25].

The superiority of the Passive Ultrasonic Irrigation + Zirconia group over the Passive Ultrasonic Irrigation + Distilled Water group was incremental rather than transformative, with the median smear layer score decreasing from 3.00 to 2.00 in the coronal third. In the present distilled water model, passive ultrasonic irrigation alone achieved measurable smear layer reduction in the coronal and middle thirds, with median smear layer scores of 3.00 compared with 4.00 for the corresponding sonic and laser control groups under identical experimental conditions. These findings suggest that passive ultrasonic irrigation contributes substantially to smear layer removal through acoustic streaming and cavitation [17]. Zirconia microparticles may provide an additional abrasive benefit [12,18]. Accordingly, the findings are consistent with a synergistic interaction in which ultrasonic activation provides the hydrodynamic conditions for particle transport and wall contact, whereas zirconia microparticles may further enhance surface debridement through physical abrasion [12,17,18]. The modest nature of the observed improvement suggests that particulate supplementation may augment, rather than replace, the mechanical action of passive ultrasonic irrigation.

### 4.2. The Anatomical Gradient and Apical Limitations

The apical third consistently exhibited the greatest resistance to smear layer removal, even when advanced activation techniques were employed. Although the overall Kruskal–Wallis test indicated significant heterogeneity among the six groups (*p* = 0.007), post hoc pairwise comparisons with Bonferroni correction revealed no statistically significant differences between any specific group pairs (*p* > 0.05). This discrepancy reflects the conservative nature of the Bonferroni adjustment, which reduces Type I error at the expense of statistical power when multiple comparisons are performed. As noted in methodological analyses of hierarchical testing procedures, a significant omnibus test does not necessarily yield significant post hoc pairwise differences, particularly when conservative corrections are applied [26]. In the apical third, anatomical constraints, including vapor lock, restricted canal volume, and energy dissipation, inherently limit irrigant exchange and particle mobilization [9,11]. These physical barriers may outweigh the effects of activation modality or particulate enhancement, such that the significant Kruskal–Wallis result likely reflects subtle distributional differences rather than clinically meaningful separation between groups. The limited irrigant volume within this confined space can further restrict bubble dynamics and compromise irrigant exchange [24]. Moreover, the close proximity of activation tips to the canal walls may promote energy dissipation, thereby reducing the effective transfer of kinetic energy to the irrigant and suspended particles [17]. Consequently, effective smear layer removal in the apical third remains highly challenging, primarily because of these anatomical constraints rather than the activation method itself [11].

### 4.3. Methodological Strengths and Clinical Implications

Interrupted activation cycles (1 min activation followed by a 10 s rest) were used as a critical control to minimize localized temperature rise. This protocol is particularly important during laser activation, as it helps protect periradicular tissues from heat-induced damage [27,28]. Additionally, standardizing tip placement at 2 mm short of the working length ensured that the procedure remained within clinically acceptable safety parameters by enhancing hydrodynamic agitation while minimizing the risk of apical irrigant extrusion [29,30].

During activation, 5 µm zirconia microparticles were added to enhance mechanical and hydrodynamic interactions with dentinal walls. Evidence from particulate-enhanced irrigation studies suggests that suspended micro- to submicron-sized particles may facilitate smear layer removal by improving surface contact and fluid agitation, with larger particles generating greater mechanical impingement on canal walls [12,18]. Dentinal tubule diameters range from approximately 1–3 µm [31]. Therefore, the 5 µm zirconia microparticles used in the present study are likely to act primarily at the tubule orifice level, promoting surface debridement while limiting deep intratubular penetration [31,32,33]. Although evaluated under ex vivo conditions, the present findings suggest that combining 5 µm zirconia microparticles with passive ultrasonic irrigation enhanced mechanical smear layer removal in the coronal and middle thirds. This observation is consistent with a synergistic mechanism: passive ultrasonic irrigation generates the hydrodynamic conditions for particle transport and wall contact, while zirconia microparticles contribute additional surface abrasion [12,17,18].

In endodontic practice, clean canal walls and patent dentinal tubule orifices are considered prerequisites for optimal sealer adaptation, as residual smear layer impedes sealer penetration and interfacial bonding [34,35]. Consistent with this concept, the present SEM-based study demonstrated that zirconia-assisted passive ultrasonic irrigation improved smear layer removal and dentinal tubule patency in the coronal and middle thirds. However, the effects of these findings on sealer adaptation, obturation quality, and radicular dentin integrity were not evaluated and remain to be established in future biomechanical and sealing outcome studies.

It is important to acknowledge that the use of distilled water as both the control irrigant and particle vehicle precludes direct comparison with 17% EDTA, the established gold standard for chemical smear layer removal. This experimental design was intentionally adopted to isolate the mechanical contribution of particle-assisted irrigation by eliminating the confounding effects of chemical demineralization. Consequently, these findings should not be interpreted as validating zirconia-assisted irrigation as a clinical alternative to EDTA-based protocols, but rather as proof-of-concept for particle-assisted mechanical debridement. Future studies should directly compare the most effective protocol identified in the present study (passive ultrasonic irrigation with zirconia microparticles) with established chelating regimens, including 17% EDTA, to determine its adjunctive clinical value.

### 4.4. Study Limitations

The absence of vital pulp tissue, periapical pressure, and organic exudates precludes full clinical replication. Additionally, the exclusive use of straight, single-rooted teeth with mature apices limits the generalizability of the findings to the curved and multi-rooted anatomies commonly encountered in clinical practice. Future studies should therefore include curved canals and multi-rooted teeth to better simulate clinical conditions and validate the present findings across a broader range of anatomical complexities.

The use of distilled water as both control and vehicle, rather than a conventional chelating agent such as 17% EDTA, precludes direct comparison with the current clinical gold standard for smear layer removal. Furthermore, the use of distilled water rather than sodium hypochlorite (NaOCl) as the irrigant vehicle distances this experimental model from clinical reality, where NaOCl is the standard antimicrobial irrigant. In addition, the stability, dispersibility, and mechanical efficacy of 5 µm zirconia microparticles in NaOCl remain unknown. Although zirconia is chemically inert and resistant to oxidation, its suspension stability, aggregation behavior, and sustained abrasive performance in alkaline NaOCl solutions have not been characterized in an endodontic setting. Whether NaOCl alters particle surface charge, promotes agglomeration, or affects dispersion uniformity requires dedicated physicochemical investigation before clinical translation can be considered.

This study evaluated smear layer removal using SEM alone and did not assess potential changes in radicular dentin integrity following zirconia microparticle activation at 45 kHz. Therefore, no conclusions can be drawn regarding the presence or absence of unwanted dentin wear under the experimental conditions used in this study. Whether this particle concentration and activation intensity produce deleterious morphological alterations remains to be determined in future studies.

Finally, this investigation evaluated a single particle dimension (5 µm); the optimal particle size and concentration for particulate-enhanced irrigation remain to be determined in future studies.

## 5. Conclusions

The incorporation of 5 µm zirconia microparticles enhanced the overall performance of the evaluated irrigation protocols, with the combination of zirconia microparticles and passive ultrasonic activation demonstrating the most favorable outcomes. This protocol achieved improved smear layer removal and tubule patency, particularly within the coronal and middle thirds of the root canal.

A distinct anatomical gradient was observed across all experimental parameters (*p* < 0.001), with the apical third consistently exhibiting the greatest resistance to smear layer removal, irrespective of the activation method or particulate enhancement. These findings suggest that regional anatomical constraints, rather than activation modality alone, remain a major determinant of debridement effectiveness.

Among the activation methods investigated, passive ultrasonic irrigation demonstrated superior mechanical cleaning performance, which may be attributed to enhanced hydrokinetic effects. Er,Cr:YSGG laser activation achieved moderate smear layer removal, whereas lower-frequency sonic activation exhibited the lowest overall debridement efficacy under the conditions of this study. Collectively, these findings support the potential of particulate-assisted irrigation, particularly when combined with passive ultrasonic irrigation, for enhancing smear layer removal.

## Figures and Tables

**Table 1:** Inter-examiner reliability for smear layer scoring using Cohen’s Kappa.

Anatomical Region	Kappa Value (κ)	Level of Agreement †	Significance (*p*)
Apical	0.822	Almost Perfect	<0.001 ***
Middle	0.751	Substantial	<0.001 ***
Coronal	0.736	Substantial	<0.001 ***

Note: *** Significant at *p* < 0.001. † Interpretation thresholds based on Landis and Koch (1977): 0.00–0.20 (Slight), 0.21–0.40 (Fair), 0.41–0.60 (Moderate), 0.61–0.80 (Substantial), and 0.81–1.00 (Almost Perfect) [21].

**Table 2:** Median and interquartile range (IQR) of smear layer scores across root canal regions (n = 10 per group).

Experimental Group	Apical Median (IQR)	Middle Median (IQR)	Coronal Median (IQR)
Sonic + Distilled Water (A1)	4.00 (0.00) ^a^	4.00 (0.00) ^b^	4.00 (1.00) ^bc^
Sonic + Zirconia (A2)	4.00 (0.00) ^a^	4.00 (1.00) ^ab^	3.00 (1.00) ^abc^
Ultrasonic + Distilled Water (B1)	4.00 (0.00) ^a^	3.00 (2.00) ^ab^	3.00 (1.00) ^ab^
Ultrasonic + Zirconia (B2)	3.00 (1.00) ^a^	3.00 (1.00) ^a^	2.00 (2.00) ^a†^
Laser + Distilled Water (C1)	4.00 (1.00) ^a^	4.00 (1.00) ^ab^	4.00 (0.00) ^c^
Laser + Zirconia (C2)	3.00 (1.00) ^a^	3.00 (0.00) ^ab^	3.00 (1.00) ^abc^

Note:Values are expressed as median (interquartile range). Within each column, different superscript letters indicate statistically significant differences between groups (*p* < 0.05), while identical letters indicate no significant difference according to the Kruskal–Wallis test followed by post hoc pairwise comparisons with Bonferroni correction. † Denotes the highest recorded smear layer removal efficacy (lowest median score) within the study.

## Data Availability

All figures, tables, and contents in this manuscript are original and were created entirely by the authors. The data supporting the findings of this study are available from the corresponding author upon reasonable request.

## Supplementary Materials

The following supporting information can be downloaded at: https://www.mdpi.com/article/doi/s1, Dataset S1: Minimal dataset used for the statistical analyses; README S1: Description of the variables included in the statistical dataset.
