# Supplementary material for: Effect of Irrigation with Zirconia Microparticles Combined with Different Activation Techniques on Smear Layer Removal: An Ex Vivo Study
Source: Dent J (Basel). 2026 Aug 7;14(8):497. doi: 10.3390/dj14080497 (PMC13510986; doi:10.3390/dj14080497)
Supplement: Supplementary file 1 [file dentistry-14-00497-s001.zip › README S1. Description of the variables included in the statistical dataset.pdf]

README – DATASET DESCRIPTION

Study Dataset Overview

This dataset accompanies the manuscript and is provided as a single Excel workbook (.xlsx). The dataset contains two sheets: Sheet 1 includes the primary raw data for 60 anonymized specimens (Tooth_ID 1–60), and Sheet 2 contains metadata and variable definitions.

Dataset Structure

The Excel workbook is organized as follows:
Sheet 1 ("Raw_Data") contains the experimental dataset including specimen identification codes, experimental group allocation, irrigant type, and smear layer scores recorded for coronal, middle, and apical thirds.
Sheet 2 ("Metadata") provides detailed descriptions of all variables, including definitions, coding information, and the scoring system used for data interpretation.

Variables Description

Tooth_ID: Unique anonymized identifier assigned to each specimen (1–60).
Experimental_Group: Allocation of specimens into different experimental groups based on irrigation and activation technique.
Irrigant_Type: Type of irrigant used, including distilled water and 5 µm zirconia suspension.
Smear Layer Score: Evaluation of smear layer using the Hülsmann scoring system (ordinal scale 1–4) applied to coronal, middle, and apical thirds.

Scoring System (Hülsmann Scale)

1 = No smear layer, dentinal tubules completely open
2 = Small amount of smear layer, partial tubule visibility
3 = Homogeneous smear layer, few tubules visible
4 = Heavy smear layer covering the entire surface

Software Used

All statistical analyses were performed using IBM SPSS Statistics software. Analyses were conducted using the graphical user interface (GUI) without the use of custom scripts or programming code.

Data Anonymization and Confidentiality

All specimens were fully anonymized using Tooth_ID codes. No patient-identifiable, personal, or confidential information is included in this dataset.

Data Completeness

The dataset includes all 60 specimens included in the study. No missing, excluded, or incomplete data were present.

Reproducibility Statement

The dataset allows full replication of all statistical analyses using standard non-parametric methods in IBM SPSS Statistics (GUI-based), without requiring external programming or syntax files.
